# Ductile bulk metallic glass by controlling structural heterogeneities

**DOI:** 10.1038/s41598-018-27285-5

**Published:** 2018-06-15

**Authors:** S. Scudino, J. J. Bian, H. Shakur Shahabi, D. Şopu, J. Sort, J. Eckert, G. Liu

**Affiliations:** 1 IFW Dresden, Institute for Complex Materials, Helmholtzstraße 20, D-01069 Dresden, Germany; 2 State Key Laboratory for Mechanical Behaviors of Materials, School of Materials Science and Engineering, Xi’an Jiaotong University, Xi’an, 710049 China; 3 Heraeus Amorphous Metals, Heraeus Deutschland GmbH & Co. KG, Heraeusstrasse 12 – 14, D-63450 Hanau, Germany; 4 Institut für Materialwissenschaft, Technische Universität Darmstadt, Otto-Berndt-Strasse 3, Darmstadt, D-64287 Germany; 5 Erich Schmid Institute of Materials Science, Austrian Academy of Sciences, Jahnstraße 12, A-8700 Leoben, Austria; 6 Departament de Física, Universitat Autònoma de Barcelona, E-08193 Bellaterra, Spain; 7 Institució Catalana de Recerca i Estudis Avançats (ICREA), Pg. Lluís Companys 23, E-08010 Barcelona, Spain; 8 Department Materials Physics, Montanuniversität Leoben, Jahnstraße 12, A-8700 Leoben, Austria

## Abstract

A prerequisite to utilize the full potential of structural heterogeneities for improving the room-temperature plastic deformation of bulk metallic glasses (BMGs) is to understand their interaction with the mechanism of shear band formation and propagation. This task requires the ability to artificially create heterogeneous microstructures with controlled morphology and orientation. Here, we analyze the effect of the designed heterogeneities generated by imprinting on the tensile mechanical behavior of the Zr_52.5_Ti_5_Cu_18_Ni_14.5_Al_10_ BMG by using experimental and computational methods. The imprinted material is elastically heterogeneous and displays anisotropic mechanical properties: strength and ductility increase with increasing the loading angle between imprints and tensile direction. This behavior occurs through shear band branching and their progressive rotation. Molecular dynamics and finite element simulations indicate that shear band branching and rotation originates at the interface between the heterogeneities, where the characteristic atomistic mechanism responsible for shear banding in a homogeneous glass is perturbed.

## Introduction

Among the current advanced materials, bulk metallic glasses (BMGs) are of significant interest for functional and structural applications due to their unique combination of properties, including high strength, unusually large elastic limit, damage tolerance that can surpass that of tough crystalline alloys and good corrosion resistance^1–3^. A limiting factor that precludes the extensive use of these attractive materials is their discrete and highly-localized plastic deformation at room temperature, which occurs via the formation and propagation of narrow shear bands^4^. Although highly malleable metallic glasses have been developed^5,6^, the macroscopic plastic deformation of BMGs is usually limited under compressive loading and is essentially zero under tension, where a single shear band propagates catastrophically soon after yielding^7–9^.

Shear bands are the carriers of plastic deformation in BMGs at room temperature; consequently, shear band multiplication, which effectively distributes the strain over several bands, is essential for attaining large plasticity under compressive or tensile loadings^7,10^. At the same time, the catastrophic propagation of a single shear band has to be limited to prevent early failure^10^. Plastic strain of BMGs can be drastically enhanced under constrained modes of loading (e.g. under compression) by using a variety of mechanical pre-treatments, which generate shear bands and create heterogeneous microstructures capable to limit their propagation^11–14^, or by the proper selection of the loading conditions^15–18^. On the other hand, only a few effective methods for improving the tensile ductility of BMGs have been developed so far. These methods, which also generate heterogeneous microstructures, include cold rolling^19,20^, high-pressure torsion^21^ and surface mechanical attrition^22^. Although the effectiveness of heterogeneous microstructures for enhancing the tensile ductility of BMGs has been established^19–22^, the limits of this approach have not been fully evaluated yet. For example, the gain of tensile plasticity in cold-rolled BMGs is rather limited^9,19,20^ because of the orientation of the pre-existing shear bands along the plane of maximum resolved shear stress, which induces plastic strain to take place exclusively via the reactivation of a few bands^23^. Exploring the full potential of these ductilization methods would thus require the optimization of the microstructural heterogeneities and, consequently, the ability to artificially create heterogeneous microstructures with designed morphology.

**Figure 1:**
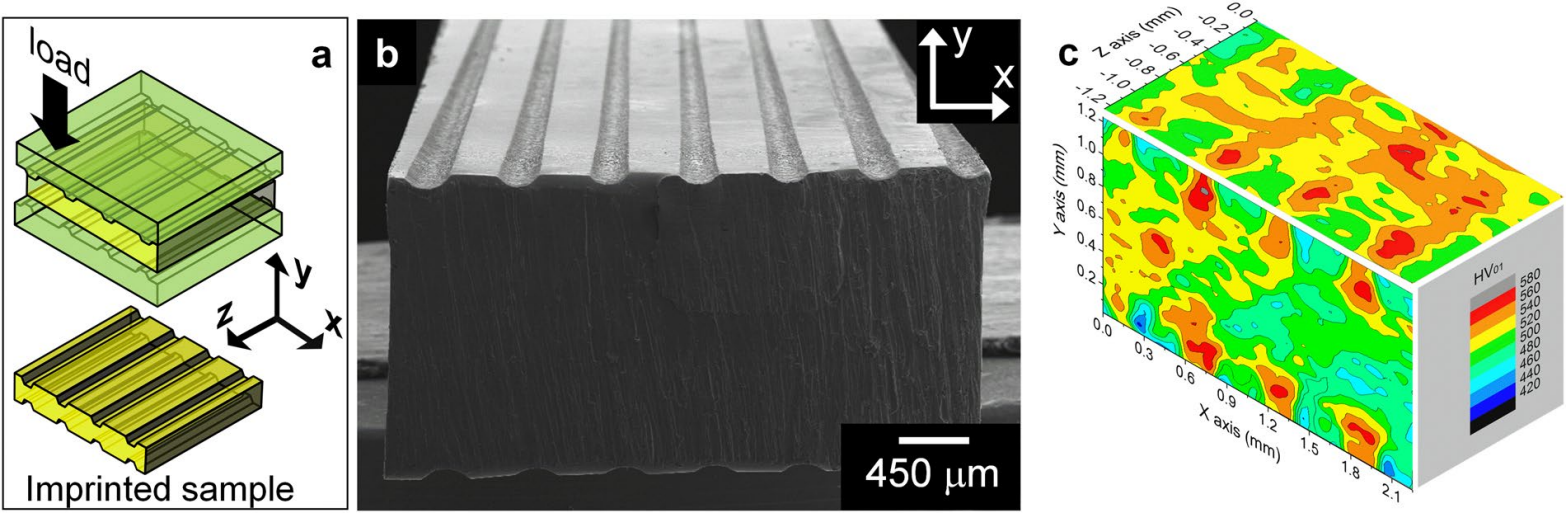
Characteristics of the imprinting process. (**a**) Schematic representation of the imprinting method and coordinate system used in this work. (**b**) Characteristic micrograph of an imprinted Zr_52.5_Ti_5_Cu_18_Ni_14.5_Al_10_ BMG showing the formation of the imprints on the sample surface. (**c**) 3D hardness map of the imprinted Zr_52.5_Ti_5_Cu_18_Ni_14.5_Al_10_ BMG.

Imprinting is a forging-like ductilization method capable to generate customized heterogeneous microstructures^24^. In this method, the metallic glass specimen (yellow parallelepiped in Fig. 1a) is placed between two imprinting tools with a regular array of linear teeth. The load is applied at room temperature along the Y axis, resulting in the generation of a linear, periodic pattern of imprints on the X-Z surface of the sample (Fig. 1b). This process is not a mere surface treatment and its effect extends well into the bulk of the sample by about 300–400 μm, where a designed heterogeneous microstructure consisting of a periodic pattern of linear, alternating hard and soft regions is created, as shown by the 3D distribution of the Vickers hardness (*HV*) in Fig. 1c. Such a heterogeneous microstructure remarkably improves the tensile ductility of the BMG by inducing shear band branching^24^. Imprinting, therefore, represents a flexible tool for the generation of designed heterogeneities with size and position suitable to be investigated at different length scales, ranging from the μm-scale events characterizing the shear bands to the structural rearrangements occurring at the atomic scale.

In contrast to the parent as-cast material, an imprinted glass is no longer homogeneous and (nominally) isotropic but it can be considered as an orthotropic material (Fig. 1c); consequently, a variation of the mechanical behavior (and shear band dynamics) with the loading angle is expected, as observed for analogous microstructures, such as for conventional composites reinforced with continuous fibers^25,26^. This gives the opportunity to study the interaction between shear bands and heterogeneities in a rather controlled manner. Accordingly, in this work the effect of the loading angle α between imprints and tensile direction on the tensile mechanical behavior of a Zr_52.5_Ti_5_Cu_18_Ni_14.5_Al_10_ BMG is analyzed. The aim of our investigation is to explore the correlation between the orientation of the heterogeneities and the changes of the mechanical properties from both experimental and computational points of view, paying special attention to the variation of the shear band morphology and identification of the characteristic (micro)structural features of the imprinted BMG in order to finally understand the mechanism responsible for the enhanced ductility.

## Results

### Microstructural variations generated by imprinting

**Figure 2:**
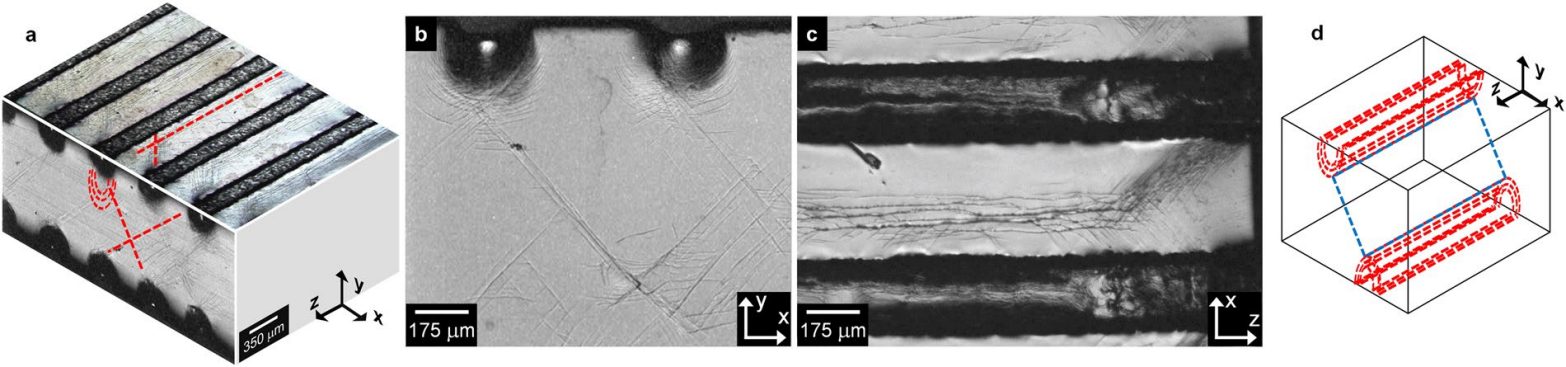
Morphology of the shear bands created by the imprinting method. (**a**) Overview of the shear band morphology for the imprinted Zr_52.5_Ti_5_Cu_18_Ni_14.5_Al_10_ BMG. (**b**) and (**c**) Shear band arrangement on the X-Y and X-Z planes, respectively. (**d**) Schematic representation of the shear band morphology in the imprinted material.

The formation of the imprints on the surface of the glass sample (Fig. 1b) occurs through the generation of shear bands. An overview of the shear band morphology is shown in Fig. 2a. On the X-Y plane, a high density of semi-circular shear bands are created beneath the imprints, as typically observed during indentation of BMGs^27^, along with shear bands forming an angle of ~45° with the Y axis (Fig. 2b). Most shear bands on the X-Z plane are approximately straight and parallel to the Z axis (Fig. 2c). Additional shear bands forming an angle of ~55° are visible near the edges of the sample and are most likely formed due to the locally inhomogeneous stress distribution. The shear bands at 55° are not visible when the imprinting is carried out on the tensile specimens because of the reduced load/contact area ratio (see Methods). Similar to the semi-circular shear bands, the density of the straight shear bands on the X-Z plane is higher near the imprints. This suggests a correlation between the shear bands on different planes to form shear planes with “half pipe” morphology parallel to the imprints (see schematic illustration in Fig. 2d) along with shear planes connecting them (blue plane in Fig. 2d).

**Figure 3:**
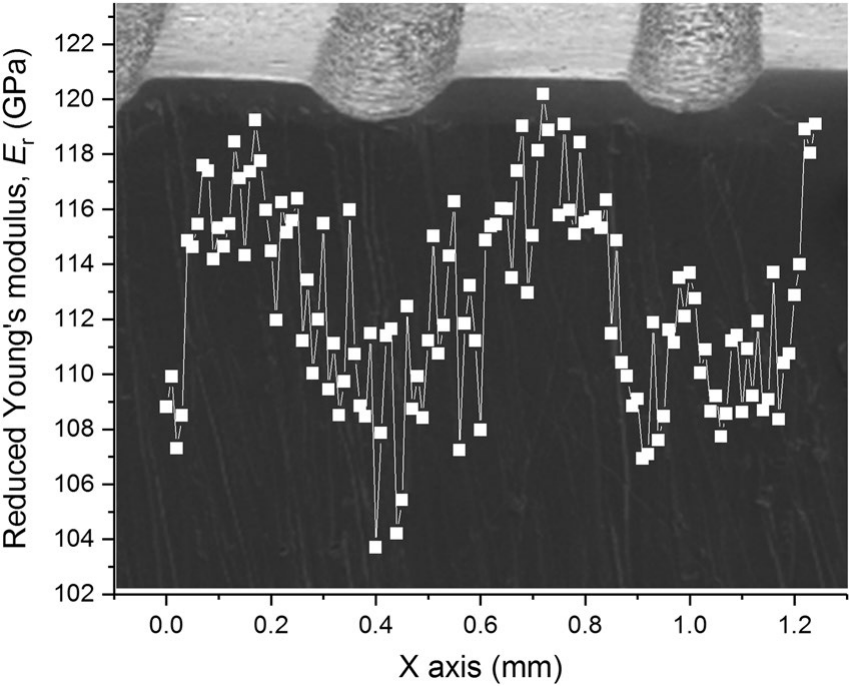
Elastic heterogeneity induced by imprinting. Variation of the reduced Young’s modulus (*E*_r_) along the X axis for the imprinted metallic glass and SEM micrograph indicating the position of the measurements with respect to the location of the imprints.

The hard and soft regions created by the imprinting process (Fig. 1c) may be characterized by different elastic properties. This is an important point of interest because the elastic mismatch arising between these regions during tensile deformation may induce stress concentrations at the hard-soft interfaces that, in turn, may influence shear band evolution. In order to clarify this aspect, the effect of imprinting on the reduced Young’s modulus (*E*_r_) of the glass was evaluated by nanoindentation. The results in Fig. 3 indicate that *E*_r_ varies periodically along the X axis with values ranging within about 105 GPa underneath the imprints, where the shear band density is higher (Fig. 2), to ~120 GPa in-between the imprints.

### Effect of heterogeneity orientation on mechanical properties

**Figure 4:**
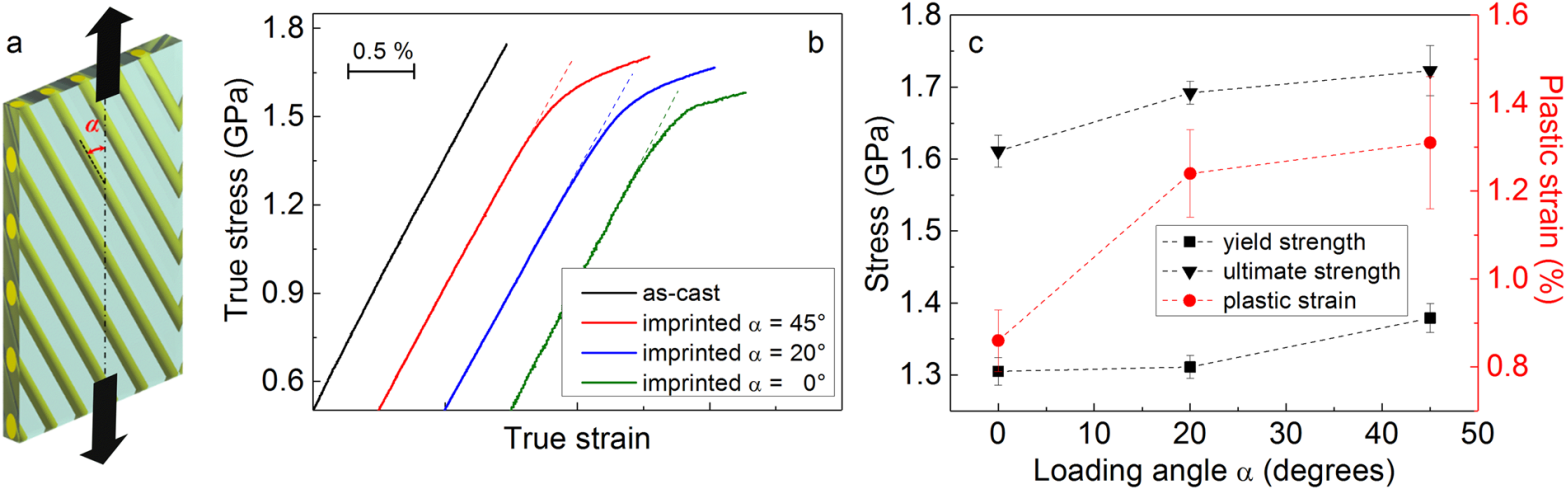
Effect of loading angle on tensile mechanical properties. (**a**) Schematic illustration of the loading angle α between imprints and tensile direction. (**b**) Room-temperature tensile stress-strain curves for the imprinted BMG for different loading angles α and (**c**) corresponding mechanical data.

The microstructure of the imprinted material is highly heterogeneous and consists of linear alternating hard and soft regions corresponding to different values of reduced Young’s modulus (Figs 1c and 3). The imprinted glass can therefore be considered as a fiber-reinforced composite made of two alternating glassy phases with the same composition but different elastic constants. The mechanical properties of fiber-reinforced composites can be tuned by varying the angle between fibers and loading direction^25,26^. We have followed this approach and explored the possibility to enhance the ductility of the Zr_52.5_Ti_5_Cu_18_Ni_14.5_Al_10_ BMG by varying the loading angle α between imprints and tensile direction (Fig. 4a).

Figures 4b,c show the tensile curves and the corresponding mechanical data of the samples with α = 0, 20 and 45° along with those of the as-cast BMG. The imprinted samples exhibit enhanced tensile ductility compared with the as-cast material, along with apparent work-hardening. Both the yield and ultimate strengths increase with increasing α, reaching values of 1.38 ± 0.02 and 1.72 ± 0.04 GPa for α = 45°. This is accompanied by an increase of plastic deformation up to 1.31 ± 0.15%. These results confirm that the use of imprinting can induce macroscopic tensile plasticity in an otherwise brittle BMG.

**Figure 5:**
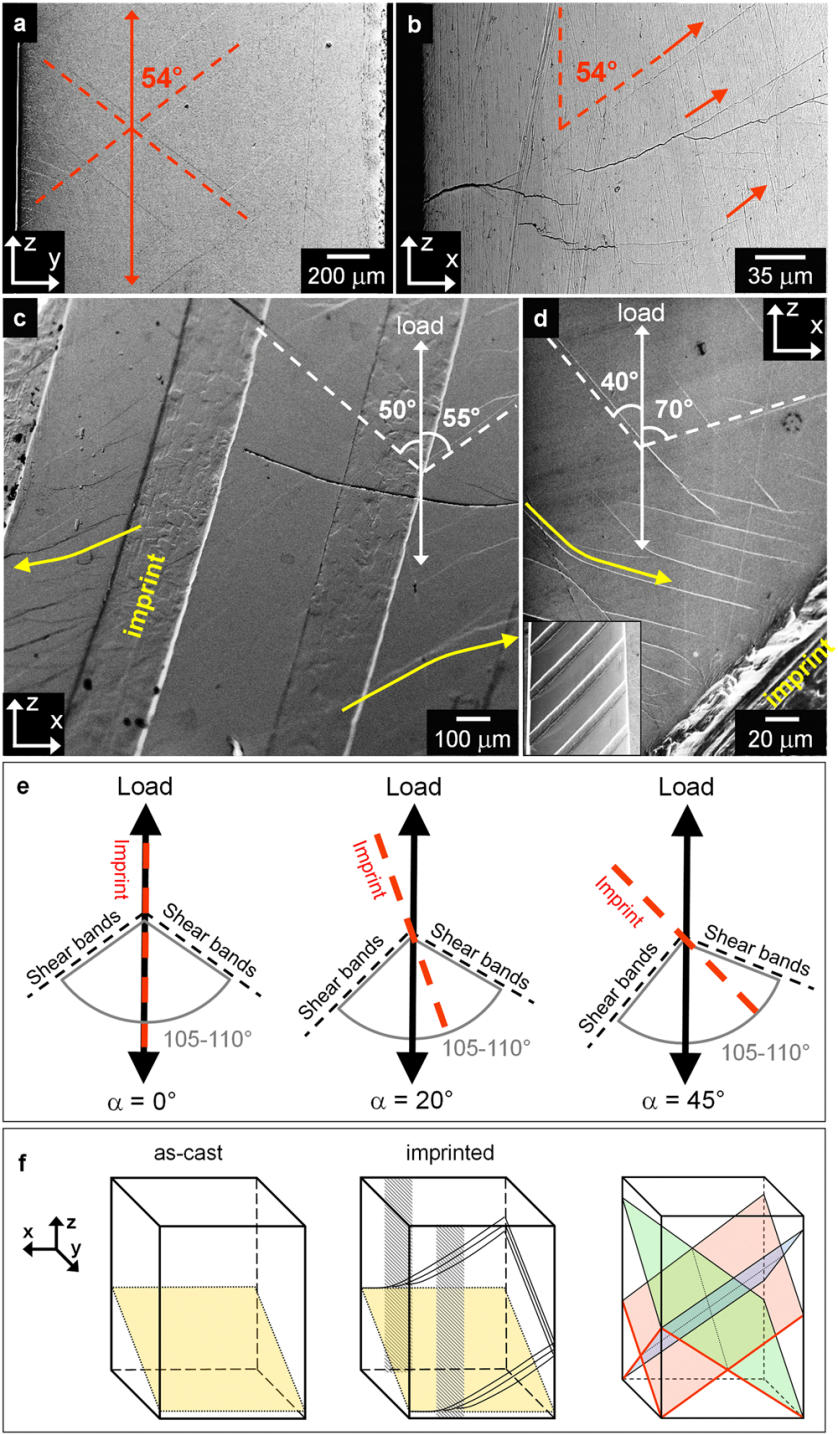
Shear band morphology after tensile tests. Shear bands on (**a**) Y-Z and (**b**) X-Z planes for the imprinted metallic glass with α = 0°. Shear bands formed on the X-Z plane for the samples with (**c**) α = 20° and (**d**) α = 45°. (**e**) Schematic representation of the rotation of the two sets of shear bands towards the direction of the imprints with increasing α. (**f**) Schematic illustration of the shear band arrangements in the as-cast and imprinted metallic glasses. The shadowed areas represent the position of the imprints and the yellow planes are the fracture planes.

After tensile tests, the imprinted glass specimens exhibit the formation of a relatively high density of shear bands, reflecting the enhanced ductility. On the Y-Z plane, the sample with α = 0° displays two symmetric families of shear bands forming an angle of ~54° with the direction of the applied load (Fig. 5a), in agreement with the range of tensile fracture angles (53–65°) reported for Zr-based BMGs^28^. When these shear bands emerge on the X-Z plane, they do not propagate at an angle of about 90°, but they are branched and deflected towards the loading direction forming again an angle of ~54° (Fig. 5b).

The orientation of the shear bands is significantly influenced by the variation of α. Although the angle between the two sets of shear bands remains rather constant (105–110°) irrespective of α, the shear bands are concomitantly rotated towards the imprints for the specimens with α = 20 and 45° (Fig. 5c–e). Furthermore, the shear bands are branched and deflected in the areas ahead of the imprints (yellow arrows in Fig. 5c,d), indicating that the shear band multiplication mechanism operating in the sample with α = 0° (Fig. 5b) is active for the different loading angles.

### Strain distribution under loading evaluated by molecular dynamics simulations

**Figure 6:**
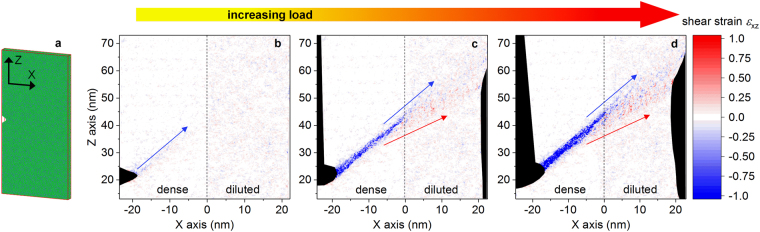
Shear banding in a simulated heterogeneous metallic glass. (**a**) Notched Cu_64_Zr_36_ sample and coordinate system used in the MD simulation. (**b**–**d**) Distribution of the atomic shear strain *ε*_xz_ with increasing load. The color code has been selected in order to highlight the largest strain values. Only half of the sample is shown in (**b**–**d**). The dashed line marks the boundary between the dense and diluted (low-density) regions.

Shear band branching, a prerequisite for achieving improved ductility, occurs ahead of the imprints, approximately at the interface between the heterogeneous regions (Fig. 5b). In order to analyze the atomistic rearrangements occurring at the interface, we have performed MD simulations of a heterogeneous Cu_64_Zr_36_ metallic glass loaded under uniaxial tension along Z (Fig. 6a). The areas underneath the imprints display a large density of shear bands (Fig. 2) and, consequently, are expected to exhibit a large amount of free volume^29^. To simulate this feature, we have generated a glassy specimen consisting of two adjacent regions: (*i*) a dense area, mimicking the regions between the imprints and (*ii*) a diluted, low-density area, representing the free volume-rich regions below the imprints. The simulation indicates that the shear band is initiated at small loads near the notch and propagates along a plane of maximum resolved shear stress (indicated by the blue arrow in Fig. 6b). At this stage, the shear strain *ε*_xz_ within the band has negative values. At higher loads (Fig. 6c), the shear band in the region comprising the interface between the dense and diluted regions is less localized. Here, the lower portion of the band is characterized by positive shear strain, implying a local change of the strain direction with respect to the part of the band having *ε*_xz_ < 0. This effect becomes stronger at higher loads (Fig. 6d), where two embryonic shear bands (indicated by the blue and red arrows) are formed.

### Stress distribution under loading evaluated by finite element simulations

Metallic glasses are elastically heterogeneous even in their as-prepared (nominally) homogeneous state^30^. The imprinted Zr_52.5_Ti_5_Cu_18_Ni_14.5_Al_10_ BMG is structurally and mechanically heterogeneous with size of the heterogeneities in the range of 100–300 μm (Fig. 1c). The heterogeneous areas in the imprinted glass exhibit different values of Young’s modulus (Fig. 3) which, as a result of the elastic mismatch arising at the interfaces between elastically-different materials^31^ (i.e. the heterogeneities), may generate a macroscopic non-uniform distribution of stress during mechanical loading. The stress/strain fields at the interfaces may then locally interfere with the mechanisms of shear band generation and propagation, leading to shear band multiplication. This interaction may also explain the observed concomitant rotation of the two sets of shear bands towards the direction of the imprints by changing the loading angle α (Fig. 5e).

**Figure 7:**
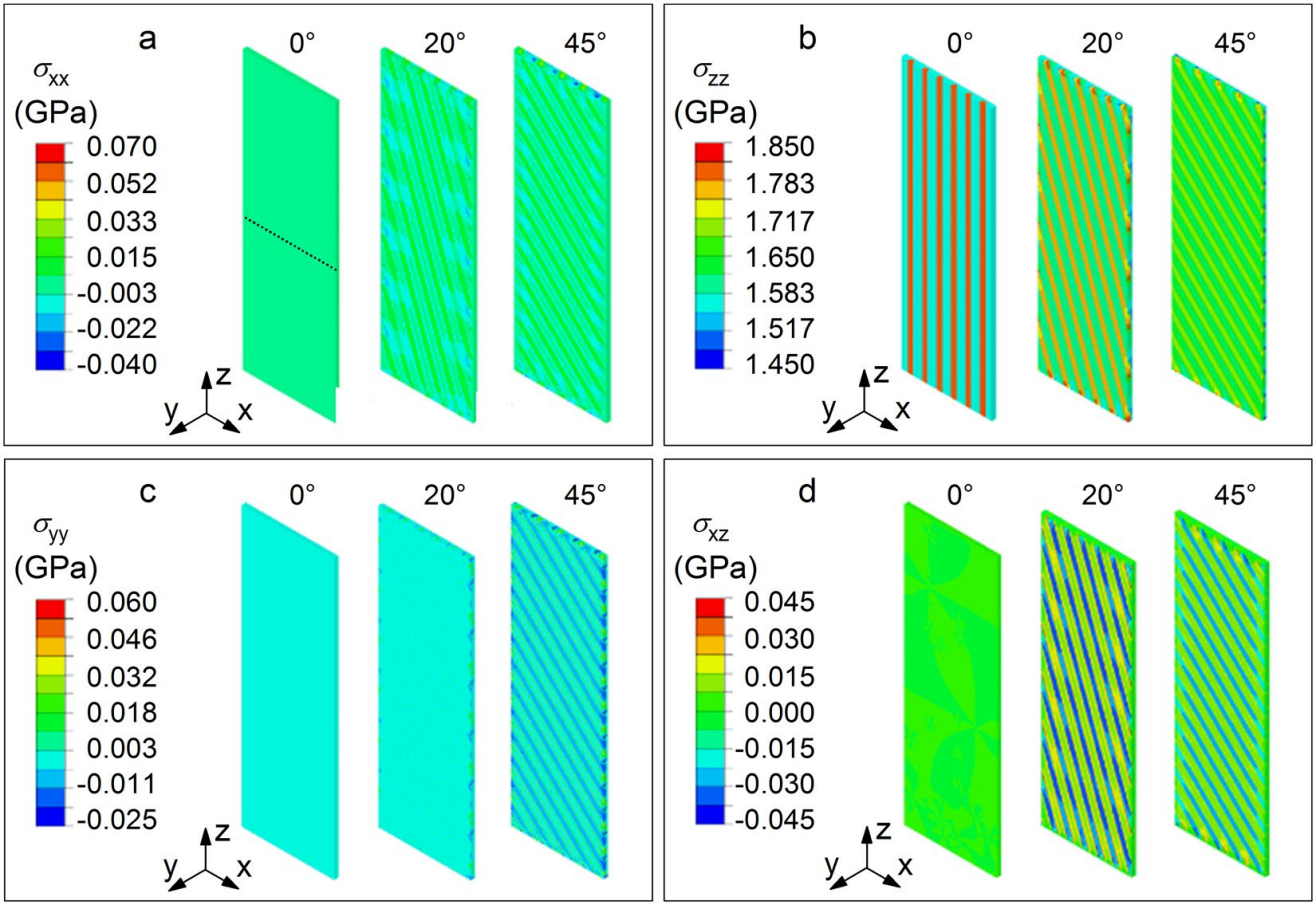
Effect of elastic heterogeneity on stress distribution. Components of the stress tensor for the imprinted material with angles α = 0, 20 and 45° modelled by FE simulations as a fiber-reinforced composite: (**a**) *σ*_*xx*_, (**b**) *σ*_*zz*_, (**c**) *σ*_*yy*_ and (**d**) *σ*_*xz*_. The figures show the stress distribution on a plane parallel to the X-Z plane and passing through the maximum diameter of the fibers.

To clarify these aspects, the stress distribution of the imprinted BMG arising in the elastic regime under uniaxial tension was investigated by FE simulations. The heterogeneous structure of the imprinted material was modeled as a fiber-reinforced composite (Fig. 4a) consisting of a continuous matrix with Young’s modulus *E* = 105 GPa and an array of parallel cylinders with *E* = 120 GPa (i.e. the lower and upper bounds evaluated by nanoindentation; Fig. 3) having different orientations (α = 0, 20 and 45°). As a characteristic distribution, Fig. 7 displays the simulated stress components *σ*_*xx*_, *σ*_*yy*_, *σ*_*zz*_ and *σ*_*xz*_ on the X-Z plane (no significant variations are observed for *σ*_*xy*_ and *σ*_*yz*_). For the sample with α = 0°, the values of *σ*_*xx*_, *σ*_*yy*_ and *σ*_*xz*_ in both matrix and fibers are extremely small and homogeneous, whereas a strong difference of the stress level between matrix and fibers occurs along the loading direction (*σ*_*zz*_). When α increases to 45°, the overall stress distribution becomes more heterogeneous, with stronger differences of *σ*_*xx*_, *σ*_*yy*_ and shear stress *σ*_*xz*_ between matrix and fibers.

**Figure 8:**
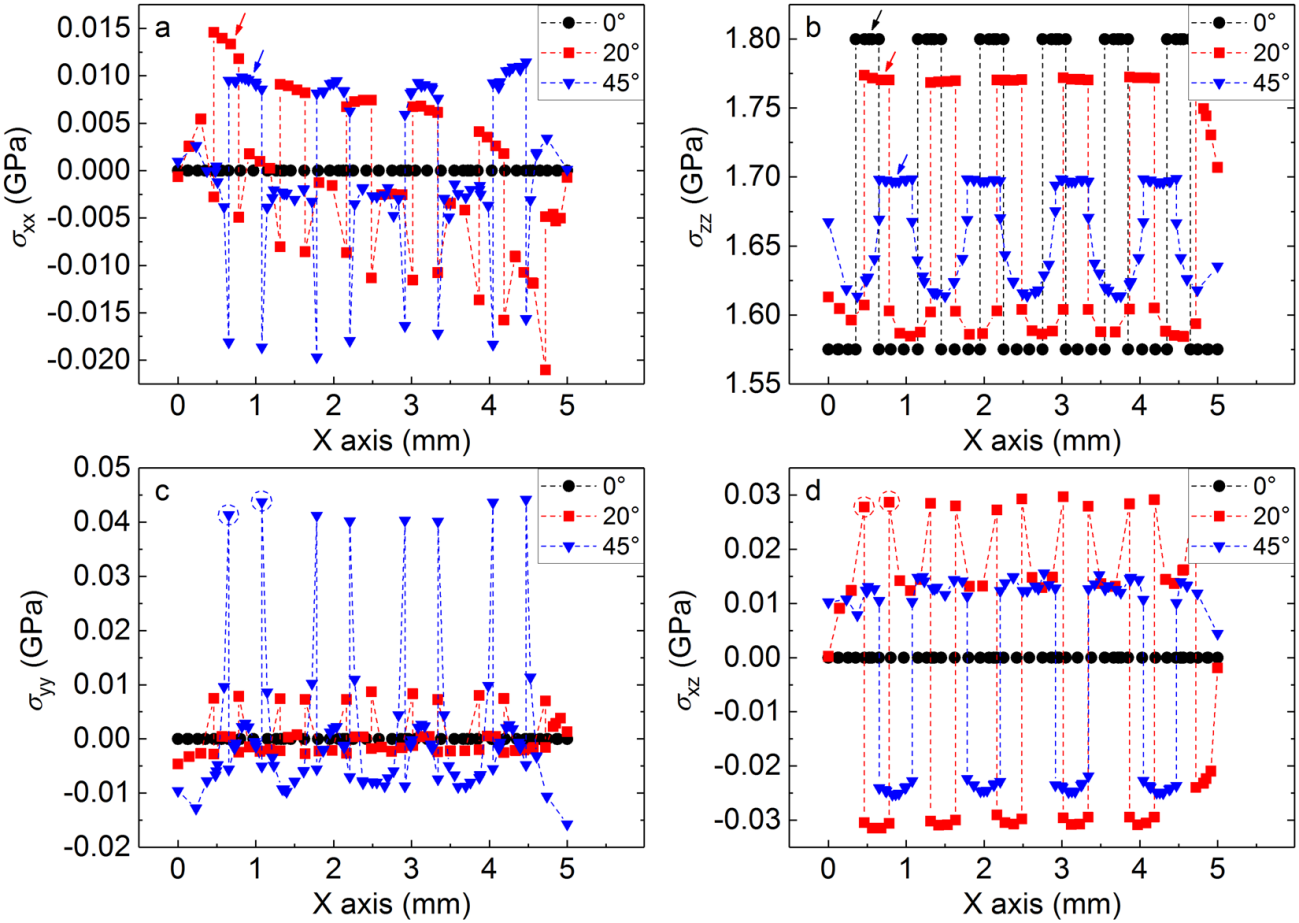
Profiles of the simulated stress components. (**a**) *σ*_*xx*_, (**b**) *σ*_*zz*_, (**c**) *σ*_*yy*_ and (**d**) *σ*_*xz*_ along the X axis for the imprinted material with angles α = 0, 20 and 45°. The position of the fibers in the different samples is indicated by arrows in (**a**) and (**b**), whereas the dashed circles in (**c**) and (**d**) mark the matrix-fiber interface.

The stress differences between matrix and fibers for the imprinted samples with α = 0, 20 and 45° can be better understood by analyzing Fig. 8, which shows the *σ*_*xx*_, *σ*_*yy*_, *σ*_*zz*_ and *σ*_*xz*_ stress profiles along the X axis (dotted line in Fig. 7a). As already observed in Fig. 7, the sample with α = 0 exhibits a negligible contribution of *σ*_*xx*_, *σ*_*yy*_ and *σ*_*xz*_, whereas the *σ*_*zz*_ component displays a sudden increase from 1.57 GPa in the matrix to 1.80 GPa in the fibers. The difference of *σ*_*zz*_ between matrix and fibers decreases with increasing α and, for α = 45°, the stress variation from matrix to fibers becomes rather gradual. The stress along the X axis (*σ*_*xx*_) is tensile within the fibers and becomes slightly compressive in the matrix; the transition is not gradual, with a relatively strong compressive stress at the interface. The (*σ*_*yy*_) component displays an opposite behavior: the interface between matrix and fibers is characterized by a strong tensile stress, which increases with increasing loading angle. Finally, the shear stress *σ*_*xz*_ shows alternating and sudden sign changes from positive values in the matrix to negative values in the fibers; the variation at the interface is particularly strong for α = 20°, where the shear stress increases from −0.03 to 0.03. The simulations thus indicate that in the different samples the stress is mainly aligned with the loading Z direction. In addition, the samples with α = 20 and 45° exhibit evident stresses along the X and Y axes. The strongest variations of normal and shear stresses for these samples occur at the matrix-fiber interface, indicating that a local change of the principal stress axes takes place here for α ≠ 0.

### Strain distribution evaluated by high-energy XRD

**Figure 9:**
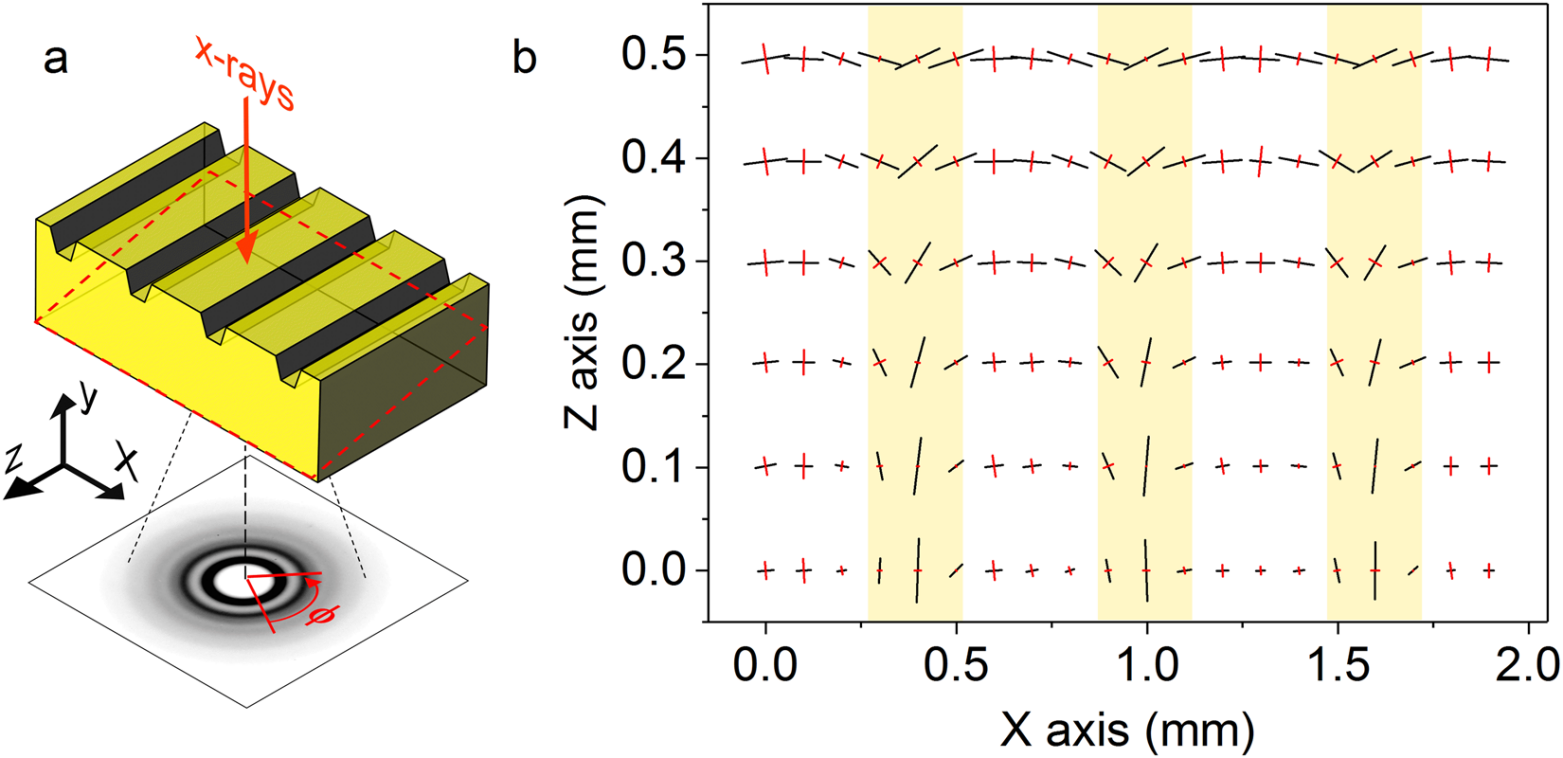
Experimental strain distribution generated by the imprinting process. (**a**) Schematic representation of the XRD setup; the dashed red box indicates the investigated area. (**b**) Eigenvectors of the strain tensor displaying the variation of the principal strain axes; the areas in yellow mark the positions of the imprints. Black vectors indicate tensile strain and red vectors compressive strain.

The FE simulations show the stress distribution that arises exclusively from the elastic mismatch characterizing the imprinted BMG. Imprinting, however, induces significant residual strain (and stress) in the material^32^, which may in turn interact with the stress concentration experienced by the material at the matrix-fiber interfaces (Fig. 8). This aspect has been investigated here by analyzing the variation of the eigenvectors of the strain tensor, which give the magnitude and direction of the principal strains. The results are presented in Fig. 9, which shows the distribution of the eigenvectors related to the imprinted BMG evaluated by high-energy XRD. The investigated area is not parallel to the X-Z plane (see dashed red box in Fig. 9a); consequently, each line of eigenvectors parallel to the X axis in Fig. 9b has a different *altitude* along the Y axis, providing a depth profile of the strain. Due to the orthotropic fiber-like symmetry of the heterogeneities (Fig. 1c), the results in Fig. 9b thus give a reasonable representation of the overall strain distribution in the imprinted glass.

The direction of the principal strains changes significantly within the imprints (highlighted in yellow in Fig. 9b), whereas it is less affected in the matrix between the imprints. In analogy with the hardness values in Fig. 1c, the strain variation depends on the position along Y: the lines of eigenvectors near the imprinted surface (e.g. z = 0.5 mm) display the strongest strain and a rotation of about 40° within the imprints. The largest strain rotation is found for the lines away from the imprinted surface (e.g. z = 0 mm); here, a variation the principal strain axes of about 90° is observed between matrix and imprinted areas.

## Discussion

Imprinting the Zr_52.5_Ti_5_Cu_18_Ni_14.5_Al_10_ metallic glass creates a heterogeneous microstructure consisting of a periodic variation of hardness and Young´s modulus (Figs 1c and 3) along with significant residual strain (Fig. 9b). Such a heterogeneous microstructure is very effective for improving the tensile ductility of the BMG through shear band branching and deflection (Fig. 5b–d). This behavior is schematically illustrated in Fig. 5f for a sample with α = 0°: while the as-cast material does not show any shear band activity except for the fracture plane at 55°, in the imprinted sample the shear bands on the Y-Z plane do not propagate on the X-Z plane at 90° with the loading axis, leading immediately to fracture as in the as-cast case, but are deflected and branched, generating a set of intersecting shear planes forming an angle of about 54° (red lines and corresponding planes in Fig. 5f). Such a shear band morphology is drastically different compared with the one observed in the as-imprinted sample (Fig. 2), indicating that plastic deformation under tension does not take place through the reactivation of the pre-existing shear bands, as it in contrast occurs for cold-rolled BMGs^23^.

In analogy with fiber-reinforced composites, the mechanical behavior of the imprinted material can be further varied by changing the loading angle α: both strength and ductility increase when α increases from 0 to 45° (Fig. 4c). This is accompanied by the concomitant rotation of the two sets of shear bands towards the direction of the imprints, as schematically illustrated in Fig. 5e. This behavior can be understood by considering the highly heterogeneous microstructure of the imprinted material and its possible interaction with the atomistic mechanism of shear band formation and propagation.

**Figure 10:**
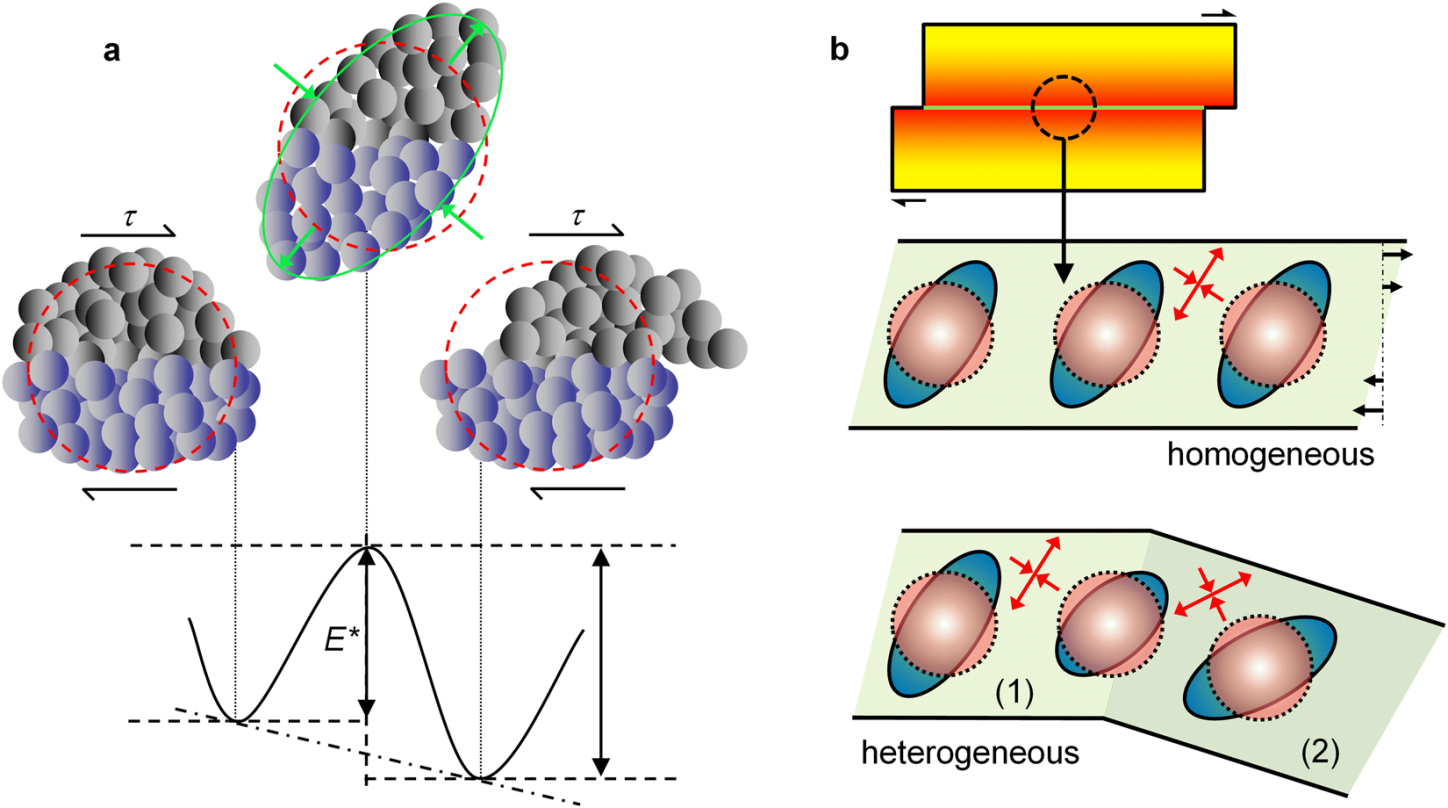
Effect of heterogeneities on atomistic mechanism of shear banding. (**a**) Schematic illustration of the mechanism of STZ activation. The red circles represent the non-activated STZs; the green ellipse and arrows indicate the activated configuration and the principal axes of deformation. (**b**) Schematic representation of a shear band and of a series of STZs in homogeneous and heterogeneous glasses. The zones marked with (1) and (2) denote areas with different degrees of residual stresses and/or stress concentration due to elastic mismatch; the local stress state is represented by red arrows.

A shear band is an approximately planar portion of material^4^ which forms by the activation and percolation of shear transformation zones (STZs), the elementary units of plasticity in metallic glasses consisting of clusters of atoms that cooperatively rearrange under the action of an applied stress^33^. The mechanism is schematically illustrated in Fig. 10a, which shows the irreversible activation of an STZ. An STZ can be described as an activated transition from one to the neighboring configurational energy minimum^34,35^. The activated configuration has higher energy (E^*^) and involves atomic rearrangement and dilatation^4^. The propensity for STZ activation is probably directional^36^. For example, easy activation of an STZ may occur when the components of the local stress coincide with the principal axes of deformation (green arrows in Fig. 10a). For a homogeneous glass, where the stress state can be assumed to be rather constant in each unit volume of material, this would lead to STZ percolation and shear band formation along a specific direction (Fig. 10b), such as under compressive loading, where shear bands are formed at an angle of about 45° with the loading direction^36,37^. On the other hand, for the present heterogeneous glass consisting of two zones with different degrees of residual strain/stress and stress concentrations arising due to elastic mismatch (Figs 7–9), the stress state might vary locally (red arrows in Fig. 10b). Under these conditions, the activation of STZs with favorable orientation in zone (1) might be progressively hindered in the transition to zone (2). Here, STZs with *easy axes* aligned with the new local stress state may be activated, causing the shear band to change direction. This effect is expected to be particularly strong in the samples with α ≠ 0, where the stress distribution during loading becomes highly heterogeneous and significant stress concentration arises at the interface of the elastically-different areas (Fig. 8), accounting for the observed change of the shear band angle with increasing α. According to this hypothesis, the activation of STZs with adverse orientation becomes progressively more difficult in the samples with α = 20 and 45°; the activation would thus require increasingly higher stress, explaining also the rise of the yield strength with α and the apparent work-hardening observed in Fig. 4.

The heterogeneous microstructure of the imprinted material not only induces deflection of shear bands, but also causes their branching (Fig. 5b). Shear band multiplication originates at the interface between the heterogeneous regions (Fig. 6). Here, the mechanism of STZ activation and percolation appears to be disturbed, the shear band broadens and finally branches. It has been reported that the plasticity of BMGs is correlated with the STZ volume^38^: metallic glasses with large STZ volume exhibit larger plasticity (and therefore higher density of shear bands) than materials with smaller volumes. The broadening of the shear band leading to branching (Fig. 6) can thus be linked to the local increase of the STZ volume. Assuming that for a given composition an STZ comprises a characteristic number of atoms, larger STZ volumes would imply a larger number of atoms involved in the cooperative rearrangement and, consequently, a more difficult STZ activation. In terms of potential energy landscape, the activation energy E^*^ (Fig. 10a) corresponding to the atomic configuration of the large STZ volume is raised and the transition becomes energetically unfavorable. Additionally, the present MD simulations reveal that the fraction of full-icosahedral (FI) clusters, which presumably form the backbone of the metallic glass structure^39^, decreases from 22% in the dense glass to about 19% in the diluted material. This reduction may perturb the STZ percolation mechanism mediated by vortexlike (rotating) structures centered on FI clusters^40^, further rising the activation energy E^*^. Plastic deformation through a single shear band with large STZ volume is thus precluded and the system explores an alternative energetically favorable path where plastic deformation is mediated by multiple shear bands with smaller STZ volumes (and smaller activation energies), as observed for the present heterogeneous imprinted BMG.

### Summary

The effect of the heterogeneities created by imprinting on the mechanical behavior under tensile loading has been investigated for the Zr_52.5_Ti_5_Cu_18_Ni_14.5_Al_10_ BMG by using both experimental and computational methods. The imprinted material is elastically and mechanically heterogeneous: Young’s modulus and hardness vary regularly with a periodicity given by the distance between imprints. The variation can be correlated with the shear band morphology, where areas with higher shear band density exhibit lower values of Young’s modulus and hardness. Additionally, significant residual strain with principal axes periodically varying across the sample can be observed for the imprinted material.

The microstructure consisting of linear and periodic heterogeneities can be considered as a composite made of two alternating glassy phases with same composition but different elastic constants and, as such, it is expected to show anisotropic mechanical properties, as observed for conventional composites reinforced with continuous fibers. We have investigated this aspect by varying the loading angle α between imprints and tensile direction. The tensile tests reveal that both strength and ductility increase when α increases from 0 to 45°. Plastic deformation does not occur through the reactivation of the pre-existing shear bands: the shear bands are branched and progressively rotated towards the direction of the imprints with increasing α.

The mechanisms leading to shear band branching and rotation have been analyzed by computational methods. MD simulations of a nanoscaled sample consisting of two adjacent dense and diluted metallic glasses indicate that shear band multiplication originates at the interface between the heterogeneous regions. The atomistic mechanism is most likely based on the increase of the STZ volume at the interface. The larger number of atoms involved in the structural rearrangement makes the STZ activation progressively more difficult, rising the activation energy of the process. Plastic deformation mediated by a single shear band with large STZ volume might then become energetically unfavorable and deformation occurs via multiple shear bands with smaller STZ volumes (and smaller activation energies). Finally, FE simulations have been used to evaluate the effect of the elastic mismatch characterizing the imprinted BMG. Again, the interface between heterogeneities appears to be also responsible for the change of the shear band direction. The results suggest that the heterogeneous microstructure (i.e residual strain/stress and stress concentrations arising at the interface during loading due to elastic mismatch) may interact with the components of the applied stress, affecting the STZ activation and percolation mechanism. STZs favorably oriented with the new local stress state may be progressively more easily activated, causing the deviation of the shear band from the direction characteristic for the homogeneous glass.

Although additional experiments are needed to explore the full potential of the imprinting process, the present findings clearly indicate that the mechanical behavior of a BMG under tensile loading can be tuned by the proper combination of the morphology of the pre-existing shear bands and (micro)structural features capable to limit their propagation. The existing thermal and mechanical ductilization methods can then be combined to achieve the optimal interaction between shear band generation and propagation. For example, discontinuous imprinting^41^ may be used to further design the morphology of the heterogeneities and the resulting shear band orientation. Furthermore, the effectiveness of the heterogeneities for inducing additional stress/strain fields during loading might be enhanced by thermal cycling^42^. Through this approach the limit of tensile brittleness characteristic of BMGs may then be overcome, finally making BMGs and their remarkable mechanical properties accessible to engineering applications.

## Methods

Plates with composition Zr_52.5_Ti_5_Cu_18_Ni_14.5_Al_10_ (at.%) and dimensions 1.7 × 35 × 40 mm^3^ were prepared by centrifugal copper mould casting. The samples for tensile tests were prepared by wire erosion into dog-bone geometry with a length of about 40 mm and a width of the testing gauge of 2 mm. Both sides of the specimens were then carefully polished to make them parallel to each other prior to imprinting. Imprinting was carried out at room temperature on the tensile specimens using a manual press and hardened steel tools with a periodic array of linear teeth. A load of 150 kN was applied on the X-Z plane for 1 minute (Fig. 1a). Specimens for tensile tests were prepared with the linear imprints forming an angle with the loading axis α = 0, 20 and 45°. Since removing the imprints on the samples surface has no significant influence on the mechanical behavior of the imprinted BMG^24^, the specimens were not grinded and polished prior to tensile testing. Tensile tests were carried out at room temperature using an INSTRON 8562 testing facility (strain rate ~1 × 10^−4^ s^−1^). The load was applied along the Z axis and the strain during mechanical tests was measured directly on the specimen using a Fiedler laser-extensometer. A minimum of four specimens for each condition was tested in order to ensure the reproducibility of the results. The onset of plastic deformation was selected at the yield point, where the strain deviates from the linearity of the elastic regime. The surface morphology of the different samples was evaluated by scanning electron microscopy (SEM) using a Gemini 1530 microscope and by optical microscopy (OM) using a Zeiss Axioskop 40. The shear band morphology of the as-imprinted material was analyzed for specimens with dimensions 1.7 × 3 × 6 mm^3^. In order to generate easily observable shear bands, in these samples imprinting was carried out at 100 kN (i.e. at higher load/contact area ratio than for the tensile specimens).

Vickers hardness maps were acquired using a computer-controlled Struers Duramin 5 Vickers hardness tester. Indents were placed every 50 μm with an applied load of 0.1 kg and a dwell time of 10 s. Nanoindentation was used to determine the reduced Young’s modulus (*E*_r_) of the imprinted BMG using a UMIS device from Fischer-Cripps Laboratories equipped with a Berkovich pyramidal-shaped diamond tip. The measurements were carried out on the X-Y plane at about 100 μm from the imprinted surface along the X axis with a spatial resolution of 10 μm. The reduced Young’s modulus was calculated using the equation^43^:

1 $${E}_{r}=\frac{1}{\beta }\frac{\pi }{2}\frac{(dF/dh)}{\sqrt{A({h}_{c})}}$$

where *dF*/*dh* is the slope of the unloading curve (i.e., contact stiffness), *β* is a geometrical constant of the order of unity, and *A*(*h*_c_) is the projected area of the indentation at the contact depth, *h*_c_.

The atomistic mechanism of shear banding was investigated in a heterogeneous Cu_64_Zr_36_ metallic glass by molecular dynamics (MD) simulations using the code LAMMPS^44^. No suitable interatomic potentials are available for the Zr_52.5_Ti_5_Cu_18_Ni_14.5_Al_10_ metallic glass; therefore, we selected the Cu_64_Zr_36_ composition because reliable Finnis-Sinclair type potential developed by Mendelev *et al*.^45^ exists. Additionally, this composition displays a high fraction of full-icosahedral clusters^39^, a prerequisite to ensure strain localization. Despite the different compositions and the largely different spatio-temporal scales, recent results have shown that the characteristic features of shear banding in the real Zr_52.5_Ti_5_Cu_18_Ni_14.5_Al_10_ BMG and simulated Cu_64_Zr_36_ glass are strikingly similar^46^; this indicates that the binary metallic glass can be used to properly simulate the behavior of the multicomponent material under tensile loading. The simulated specimen was generated as follows. First, a configuration of 8000 atoms is brought into the glassy state by quenching it from the melt to 50 K with a cooling rate of 10^10^ K/s at zero pressure. A rectangular-shaped sample with dimensions 46 × 5 × 106 nm^3^ was then created by replicating the initial glass cubic cell, giving a total number of atoms of about 1.5 × 10^6^. To emulate the two-phase (hard and soft) structure and, consequently, to simulate the shear band behavior at the hard-soft interface, we randomly remove 1% of atoms from half sample. 1% dilution rejuvenates the glass structure and, at the same time, ensures localization of the plastic deformation (not shown here). After dilution, the structures is relaxed to zero pressure at 50 K for 100 ps and most of the excess free volume is trapped within the diluted structure. This creates a composite structure consisting of two metallic glasses with dense/hard and dilute/soft structures having interface parallel to the Z axis. In order to control the position and the propagation path of the shear band, a stress concentrator (notch) was created at the surface of the specimen on the hard, dense side. The notched sample was loaded under uniaxial tension along Z at a low, constant temperature of 50 K using a constant strain rate of 4 × 10^7^ s^−1^. Periodic boundary conditions were applied along the Y and Z directions, while free surface conditions were used along the X axis. The Green-Lagrangian strain tensor was calculated and visualized using the OVITO software^47^.

Finite element (FE) simulations were performed to investigate the elastic behavior and the stress distributions of the imprinted BMG under uniaxial tensile loading. The commercial FE software Abaqus was utilized here. The dimensions of the simulated specimens were 5^*x*^ × 0.5^*y*^ × 15^*z*^ mm^3^. The heterogeneous structure of the imprinted material was modeled as a fiber-reinforced composite consisting of a continuous matrix with Young’s modulus *E* = 105 GPa and an array of parallel cylinders with diameter of 0.3 mm, spacing of 0.5 mm and *E* = 120 GPa. The Poisson’s ratio used for the calculations was 0.31^48^. In the simulations, both fiber and matrix were modeled as perfectly elastic materials. Quadratic tetrahedron elements were selected for meshing the simulated sample, with a minimum number of elements of ~1.3 × 10^4^. To apply uniaxial tensile loading, simple supported boundary conditions were applied at one end of the sample, and uniform displacement was applied at the other end. The maximum engineering strain of the simulation was ~1.5%. In order to evaluate the influence of the fibers orientation and to replicate the conditions experimentally met in the imprinted specimens, the simulations were performed using different loading angles α = 0, 20 and 45°. The load was applied along the Z axis.

The structure of the samples was studied by X-ray diffraction (XRD) in transmission geometry using a high-intensity high-energy monochromatic synchrotron beam (λ = 0.0125 nm) at the ID11 beamline of the European Synchrotron Radiation Facilities (ESRF). Specimens of the as-cast and imprinted samples with uniform thickness of 100 μm were scanned over an area of 0.5 × 1.8 mm^2^ using a beam size of 50 × 50 μm^2^, which was thus smaller than the heterogeneous regions (Fig. 1c). Diffraction patterns were collected using a two-dimensional charge coupled device (CCD) camera^49^ every 100 μm along the X axis in a series of six parallel arrays with a spacing of 100 μm along Z. For comparison purposes, as-cast homogeneous samples were analyzed by XRD using the same parameters as used for the imprinted glass. The two-dimensional patterns were then integrated in 10° azimuthal slices between 0 and 360°using the Fit2D program^50^ to give the XRD intensity distributions *I*(*q*, *ϕ*_*i*_) as a function of the scattering vector *q* and azimuthal angle *ϕ*_*j*_ (*j* = 10…360°). The strain *ε* was measured through the shift of the first scattering maximum (*q*_1_) of the imprinted material with respect to the as-cast glass as

2 $$\varepsilon =\frac{{q}_{1}^{ac}-{q}_{1}^{imp}}{{q}_{1}^{imp}}$$

where ${q}_{1}^{ac}$ and ${q}_{1}^{imp}$ are the positions of the first maximum for the as-cast and imprinted samples. The position of *q*_1_, which can be reliably used to analyze the structural changes taking place in the medium-range order of the glassy structure^51^, was evaluated by fitting using a pseudo-Voigt function. The three components of the strain tensor (*ε*_*xx*_, *ε*_*zz*_ and *ε*_*xz*_) for each point scanned on the samples were determined according to the method described in Poulsen *et al*.^52^. No distinction is made here between elastic and plastic strain, and the term strain is used in a general sense simply representing the variations occurring in the relative positions of the particles forming a body^53^.
